# Core–Shell UCNP@MOF Nanoplatforms for Dual Stimuli-Responsive Doxorubicin Release

**DOI:** 10.1021/acsabm.4c01796

**Published:** 2025-04-09

**Authors:** Marina P. Abuçafy, Beatriz B. S. Ramin, Angelica E. Graminha, Willy G. Santos, Regina C. G. Frem, Adelino V. G. Netto, José Clayston M. Pereira, Sidney J. L. Ribeiro

**Affiliations:** † Institute of Chemistry, São Paulo State University, Araraquara, São Paulo 14800-060, Brazil; ‡ Federal University of ABC, UFABC, Santo André, São Paulo 09210-170, Brazil

**Keywords:** multifunctional nanocarrier systems, metal−organic frameworks, upconversion nanoparticles, pH-sensitive drug release, laser stimulation

## Abstract

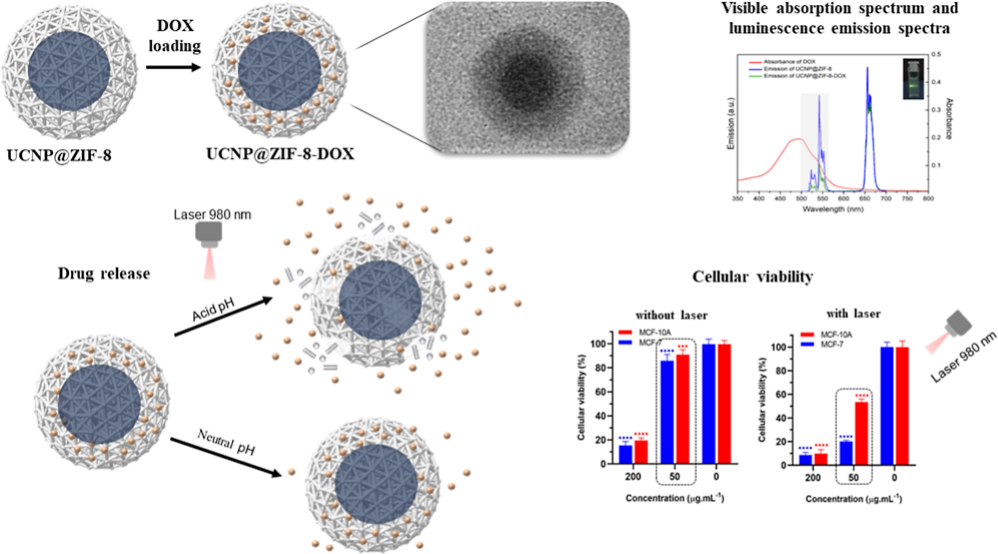

Nanocarrier systems with multifunctional capabilities hold great potential for targeted cancer therapy, particularly for breast cancer treatment. Metal–organic frameworks (MOFs) are notable for their high porosity and, in some cases, biocompatibility, with ZIF-8 being particularly advantageous due to its pH-sensitive degradability, enabling selective drug release in tumor environments. Additionally, lanthanide-doped upconversion nanoparticles (UCNPs) offer unique optical properties that enhance both imaging and therapeutic applications. In this study, NaYF_4_/Yb^3+^Er^3+^ UCNPs were synthesized via a hydrothermal method, subsequently coated with poly(acrylic acid) (PAA) and encapsulated within a ZIF-8 shell, forming of UCNP@ZIF-8 core–shell nanocomposites. This system was designed to leverage stimulation by a 980 nm laser and acidic pH to facilitate drug release. When exposed to this specific laser wavelength, the nanocomposites demonstrated significantly enhanced drug release, achieving up to 90% release of the incorporated antitumor drug, doxorubicin (DOX), in acidic environments. In vitro studies indicated selective cytotoxicity, with MCF-7 tumor cell viability decreasing from 85.7% to 20% following laser activation, while showing minimal toxicity toward healthy cells. These findings underscore the potential of the UCNP@ZIF-8 nanocarrier system as a pH and laser-responsive platform for improved cancer therapy, enabling precise control over drug delivery while minimizing side effects on surrounding healthy tissues.

## 1 Introduction

Multifunctional nanocarrier systems have gained attention as promising tools for cancer therapy such as breast cancer, which is one of the most prevalent forms of malignant neoplasms globally. Chemical compounds based on carrier systems may be developed through synergistic integration between therapeutic agents and nanomaterials that enhance biological functionalities such as targeted delivery, controlled release, and sensitivity to external stimuli.^1^

Metal–organic frameworks (MOFs) have attracted substantial interest due to their distinctive properties among the diverse range of nanocarriers. MOFs are highly porous extended structures formed by metal ions or metallic clusters coordinated with multitopic organic ligands, offering very high surface areas, adjustable pore sizes, and diverse possibilities of chemical modification of the MOF structure. These characteristics render MOFs particularly well-suited for applications as drug delivery systems.^2^ Consequently, advancing multifunctional systems based on Metal–Organic Frameworks (MOFs) have garnered significant attention in the global scientific community.^3^ Among these, Zeolitic Imidazolate Frameworks (ZIFs),^4^ a subclass of MOFs are particularly noteworthy due to their topological similarity to zeolites, characterized by high porosity and chemical selectivity, where metal ions and imidazolate-based organic ligands replace silicon and oxygen, respectively.^5^ ZIF-8, in particular, has shown considerable potential as a drug delivery system due to its green synthesis and also high surface area, robust structure, exceptional stability, biocompatibility, and ease of functionalization. Besides that, its pH-sensitive degradability also makes ZIF-8 nanoparticles excellent candidates for selective drug release in the acidic microenvironment of tumors.^6^

In addition to new chemical structures with optical properties, lanthanide-doped upconversion nanoparticles (UCNPs) have attracted significant attention across nanomedicine.^7^ UCNPs are particularly noteworthy for their ability to convert two or more low energetic photons from near-infrared (NIR) region to a higher energetic photon, which may be located in the therapeutic window region, providing more deep tissue penetration for treating cancers in internal organs.^8^ This feature makes UCNPs a promising alternative to photodynamic therapy, photochemical redox processes, and various imaging modalities, which include fluorescence imaging, X-ray computed tomography (CT), and magnetic resonance imaging (MRI).^9^

It is well-known that UCNPs based on rare earth elements are usually non-biocompatible systems. Nevertheless, after chemical modification of the nanoparticle’s surface, a range of biocompatible and functional UCNPs has been developed for potential pharmaceutical applications.^10^ However, most studies using UCNPs for biological applications involve incorporating them into matrices such as mesoporous silica through physical or chemical interactions.^11^ Unfortunately, these materials often diminish the luminescent properties of UCNPs. Therefore, developing new composites that utilize UCNPs for bioimaging or drug delivery is necessary. Combining UCNPs with MOFs, such as ZIF-8, offers a promising approach for novel and distinct theranostic systems.

Herein, an UCNP@ZIF-8 core–shell nanocomposite was synthesized using a one-pot methodology to produce a hybrid nanomaterial combining the porosity of MOFs with the optical properties of UCNPs. One of the main challenges in MOF-based drug delivery systems is achieving precise control over drug release under physiological conditions. In this work, we address these challenges by exploring the dual-stimuli-responsive release of doxorubicin (DOX), triggered by both pH and NIR laser irradiation, to enhance therapeutic efficiency. The findings contribute to the development of MOF-based drug delivery platforms with improved control over drug release kinetics, expanding the potential of these hybrid nanomaterials in cancer therapy.

## 2 Experimental Section

### 2.1 Synthesis of PAA-Coated UCNPs

UCNPs based on NaYF_4_/Yb^3+^Er^3+^ nanoparticles were synthesized according to a previously reported hydrothermal method.^12^ Briefly Y(NO_3_)_3_·6H_2_O (1.66 mmol), Yb(NO_3_)_3_·5H_2_O (0.46 mmol), and Er(NO_3_)_3_·5H_2_O (0.08 mmol) were mixed with sodium citrate (1.2 mmol) under vigorous stirring at room temperature for 30 min. Then, 3 mL of DI water, 23 mL of ethanol, and 150 mg of Cetyltrimethylammonium Bromide (CTAB) were added, followed by a dropwise addition of sodium fluoride (16.0 mmol) while stirring for 2 h. After adding 1.5 mL of nitric acid, the solution was transferred to a 100 mL Teflon-lined autoclave and incubated at 180 °C for 12 h.^13^

To coat the nanoparticles with poly(acrylic acid) (PAA), 143 mg of PAA (*M*_W_ = 1800) was solubilized in deionized water and the pH was adjusted to 8. The UCNP dispersion was added dropwise and stirred for 5 h. Subsequently, PEG (10 mL) was added to the UCNP@PAA mixture, which was stirred at 105 °C for 1 h, followed by incubation in a Teflon-lined autoclave at 160 °C for 2 h.^13^

### 2.2 Synthesis of ZIF-8 Nanoparticles

ZIF-8 nanoparticles were synthesized following the chemical methodology proposed by Cravillon and co-workers.^14^ An aqueous solution of Zn(NO_3_)_2_·6H_2_O (0.25 mol L^–1^) was rapidly mixed with an aqueous solution of 2-methylimidazole (2-MeIM) (2.0 mol L^–1^) under magnetic stirring for 1 h at room temperature. After the reaction, ZIF-8 crystals (MOF structures) were obtained, isolated by centrifugation (4000 rpm, 10 min) and washed with ultrapure water to remove the initial reactants. The sample was dried in a vacuum oven at 50 °C for 24 h.

### 2.3 Preparation of UCNP@ZIF-8 Core–Shell Nanoparticles

The core–shell structure was formed with the previously synthesized PAA-capped UCNP using a one-pot method. The UCNPs were dispersed in water, and 9.87 mmol of zinc nitrate was added and stirred for 1 h to allow interaction between Zn^2+^ and the PAA on the UCNP surface. Subsequently, 79.04 mmol of 2-methylimidazole (2-MeIM) was added dropwise, and the mixture was stirred for 1 h at room temperature.

After each synthesis step, the nanoparticles were collected by centrifugation and washed with deionized water and ethanol.

### 2.4 Physicochemical Characterization

X-ray diffraction (XRD) was carried out to evaluate the crystal states of samples using an X-diffractometer SmartLab SE (Rigaku, Neu-Isenburg, Germany). All samples were measured at 60 mA and 40 kV, with a scan range of 5–80°, with Cu Kα radiation (λ = 1.5418 Å), the scan speed of 1 s/step, and a step size of 0.05°. Scanning electron microscopy (SEM) was carried out to evaluate the morphology of nanoparticles. The samples were carbon-coated to about 5 nm thickness using a Sample Sputter Coater SCD 050 (Bal-Tec, Wallruf, Germany). TEM images were taken on a scanning electron microscopy JEOL-JEM2100. Fourier transform infrared FTIR spectra were acquired, at room temperature, between 4000 and 400 cm^–1^ using a Thermo Scientific Nicolet IS5. Zeta potential (ξ) value was analyzed on a Zetasier Nano ZS photon correlation spectroscopy (Malvern Instruments, Malvern, UK), and measurements were performed in triplicate from fresh nanocrystal suspensions at room temperature (25 °C). N_2_ sorption isotherms were obtained at 77 K using an ASAP porosimeter (accelerated surface area and porosimeter system, model 2013; Micromeritic, Norcross, GA, USA). The upconversion luminescence emission spectra were recorded on a Horiba Jobin Yvon spectrofluorometer (model fluorolog-3 FL3–122) equipped with a photomultiplier tube (model R 928 P, Spex) sensitive from 185 to 900 nm.

### 2.5 Drug Encapsulation and Release Studies

The UCNP@ZIF-8-DOX system was synthesized by adding DOX (2 g L^–1^) zinc nitrate (0.25 mol L^–1^) and UCNP suspension (0.33 g L^–1^) suspension, stirring for 1 h. After that, the 2-MeIM aqueous solution (2.0 mol L^–1^) was added dropwise to facilitate the MOF formation on the UCNP surface. The final product UCNP@ZIF-8-DOX system was washed with ultrapure water to eliminate the reactant products of synthesis.

To quantify the encapsulation efficiency (EE %), an analytical curve of DOX was established using UV–vis (Cary 60 Spectrophotometer, Agilent, Australia) at 490 nm, which allowed for the indirect determination of free drug concentration in the supernatant. The drug loading efficiency (LE %) and drug loading capacity (LC) were measured according to the following eqs 1 and 2, respectively
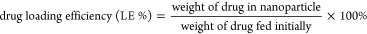
1
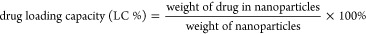
2

To measure the effects of pH changes in the DOX release, the UCNP@ZIF-8-DOX was immersed in an aqueous solution at 37 °C, pH = 7.4 or pH = 5.0, using 0.1 mol L^–1^ phosphate-buffered. These pH conditions were selected to simulate health and tumor cell environments, respectively. The samples were taken from the medium at each time point and replaced with fresh buffer to maintain sink condition. Some samples were irradiated with a 980 nm NIR laser using a power density of 0.8 w cm^–2^ for 5 min, with the concentration of DOX measured by absorbance spectroscopy in triplicate.

### 2.6 Cell Culture, Viability Evaluation, and Cellular Analysis

The cell viability evaluation was performed using the breast tumor human cell lines SK-BR-3 (ATCC-HTB-30), MCF-7 (ATCC-HTB-22), and nontumoral cell line MCF-10A (ATCC-CRL-10317). The cells were routinely maintained at 37 °C in a humidified 5% CO_2_ in RPMI 1640 (SK-BR-3 and MCF7) containing fetal bovine serum (FBS) 10% supplemented, and DMEM/F12 medium containing horse serum (HS) 5%, EGF (0.02 mg mL^–1^); hydrocortisone (0.05 mg mL^–1^); cholera toxin (0.001 mg mL^–1^) and insulin (0.01 mg mL^–1^) (MCF-10A). All media contained penicillin (100 UI mL^–1^), and streptomycin (100 mg mL^–1^).

The effect of irradiation on a sample was also evaluated, where after 4 h of treatment the cells were irradiated in 980 nm laser at a power density of 0.8 W cm^–2^ for 5 min. Cell cytotoxicity was measured after 48 h of treatment using the absorbance of the conversion of MTT, [3-(4,5-dimethylthiozol-2-yl)-2,5-diphenyltetrazolium bromide] to formazan by metabolically viable cells was read on a plate reader (Synergy-HTX) at a wavelength of 570 nm, according to Mosmann.^15^ Statistical analysis was performed to determine the inhibitory concentrations of 50% viability (IC_50_) using Hill’s equation in GraphPad Prism 9.0 software, in which cell viability was expressed as a percentage relative to the negative control.

Additionally, exponentially growing MCF-7 and MCF-10A cells were collected, counted, and seeded (5 × 10^4^ cells mL^–1^) and plated on 12-well plates. Cells were allowed to grow overnight at 37 °C and 5% CO_2_ atmosphere and then, treated or not with different concentrations of the samples DOX-UCNP@ZIF-8 for 48 h. Cell morphology was observed in an inverted microscope (Labomed-TCM 400) with an amplification of 100× and images were captured with a camera (Labomed-iVu 5100 CMOS).

For the colony formation assay, adherent cells were treated with DOX-UCNP@ZIF-8 (50 and 25 μg mL^–1^) for 48 h, after which the medium was replaced with a fresh medium devoid of nanoparticles. Following a 10 day incubation, the cells were rinsed with methanol and acetic acid (3:1) for 5 min and stained with methanol and 0.5% crystal violet for 30 min. Colonies were analyzed for number and size using ImageJ software, with plate efficiency calculated using eq 3^4^
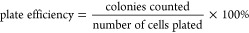
3

### 2.7 Statistical Analysis

The statistical analysis of the relative cell viability percentage data was conducted by using Prism 8.0 software. The Kruskal–Wallis test, followed by Dunnett multiple comparison test, was employed for the analysis. In all statistical evaluations, a significance level of *p ≤ 0.05 was utilized to determine the presence of significant differences between the control and treatment groups.

### 2.8 Theoretical Calculation

The electronic properties of the studied DOX were investigated using Density functional theory (DFT) methods via utilizing B3LYP level with 6-31G+(d,p) basis set. An optimized single-point calculation was conducted using the DOX structure obtained from crystallographic data reported in the literature.^16^ Molecular electronic properties such as HOMO, LUMO levels, and electrostatic potential are obtained and presented by utilizing the Gaussian 09 program.

## 3 Results and Discussion

### 3.1 Synthesis and Characterization of the Core–Shell UCNP@ZIF-8 System

**Figure 1:**
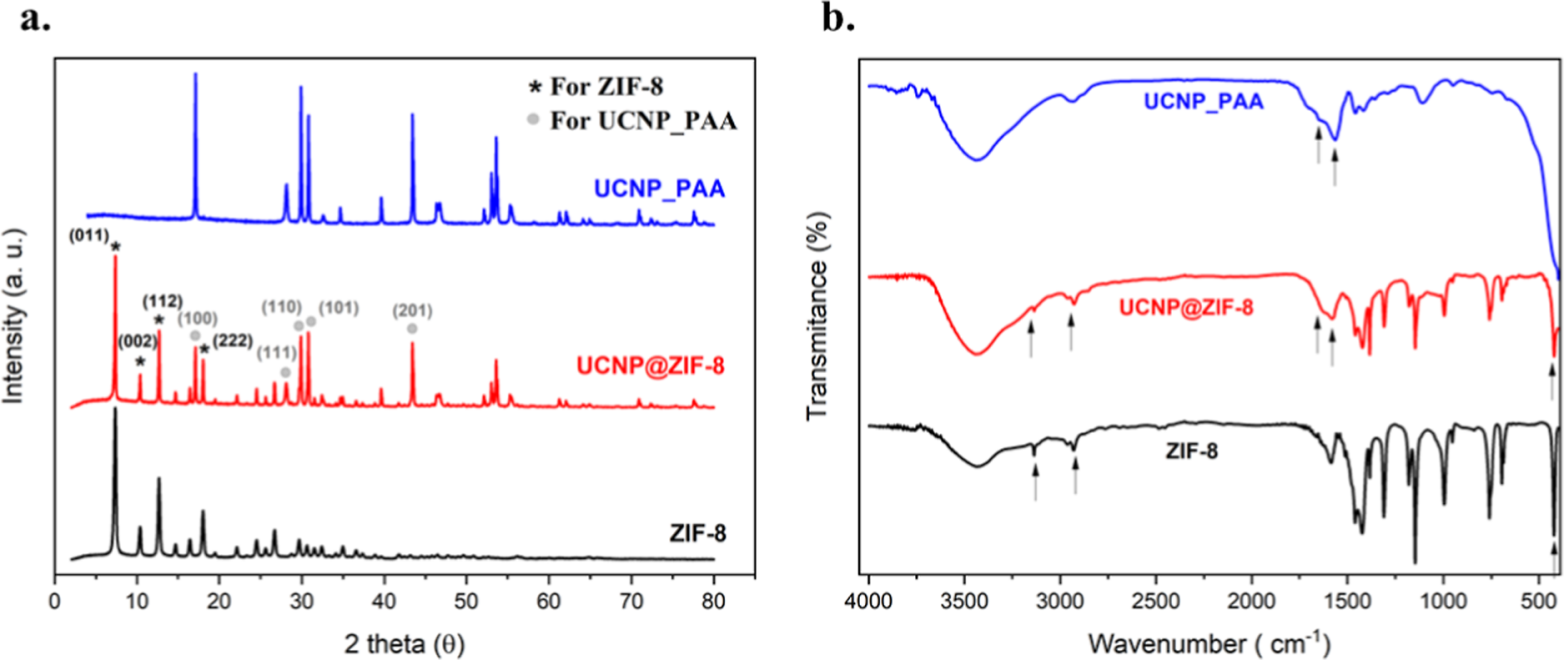
(a) X-ray diffraction patterns and (b) FT-IR spectra of ZIF-8, UCNP_PAA, and UCNP@ZIF-8.

The X-ray diffraction pattern of UCNP@ZIF-8 composite was determined and compared with those obtained for UCNPs and ZIF-8 pristine materials, as shown in Figure 1a. The X-ray diffractogram of the UCNPs modified with PAA confirms the crystalline structure of the synthesized UCNPs. The diffraction peaks at 17.02°, 28.17°, 29.88°, 31.17°, and 43.59° (2θ values) can be attributed to (100), (111), (101), and (201) planes, which strongly indicate the cubic phase for the NaYF_4_/Yb^3+^, Er^3+^.^17^ In addition to the characteristic UCNPs peaks, the diffractogram of UCNP@ZIF-8 also exhibits peaks at 7.34°, 10.41°, 12.73°, and 18.07° (2θ values), corresponding to planes (011), (002), (112), and (222), of ZIF-8^4^ structure, confirming its crystallinity. No evidence of phase change in the formation of the nanoparticulated system was observed after the formation of the UCNP@ZIF-8 or UCNP@ZIF-8-DOX systems.

Figure 1b shows the Fourier transform infrared (FT-IR) spectra of UCNP_PAA, ZIF-8, and UCNP@ZIF-8. The FT-IR spectrum of UCNPs UCNPs modified with PAA shows a band at 1567 cm^–1^, attributed to −COO^–^ asymmetric stretching on the UCNP surface, and a band at 1660 cm^–1^, associated with the –CO stretching of the PAA ligand.^18^ Furthermore, several bands corresponding to ZIF-8 were observed in the spectrum of UCNP@ZIF-8. The bands at 2929 and 3135 cm^–1^ were assigned to the aromatic and aliphatic C–H stretch of the imidazole, respectively.^19^ The band at 422 cm^–1^ was attributed to the Zn–N metal–ligand stretching mode, while bands in the region of 1100–1400 cm^–1^ were associated with C–N stretching modes.^20^ Thus, the infrared vibrational spectra show characteristic bands of both ZIF-8 and UCNP, indicating that their chemical structure was retained after the formation of the core–shell structure.

Figure S1 of the Supporting Information shows the zeta potential values obtained by dynamic light scattering (DLS) for UCNP_PAA, ZIF-8, and UCNP@ZIF-8. The zeta potential value obtained for the pristine ZIF-8 was +35.7 mV, while that of UCNP_PAA nanoparticles was −18.8 mV due to the presence of anionic carboxylate groups on the nanoparticle surface. The positive charge within the ZIF-8 structure promotes the electrostatic adsorption of negatively charged PAA molecules, resulting in the formation of the UCNP_PAA composite. This composite has a negative surface charge, which facilitates the electrostatic binding and the formation of the UCNP@ZIF-8 composite. The negative charge of the composite particles (−11 mV) forms a repulsive force between composite units, stabilizing the nanocomposite size and chemical structure.

**Figure 2:**
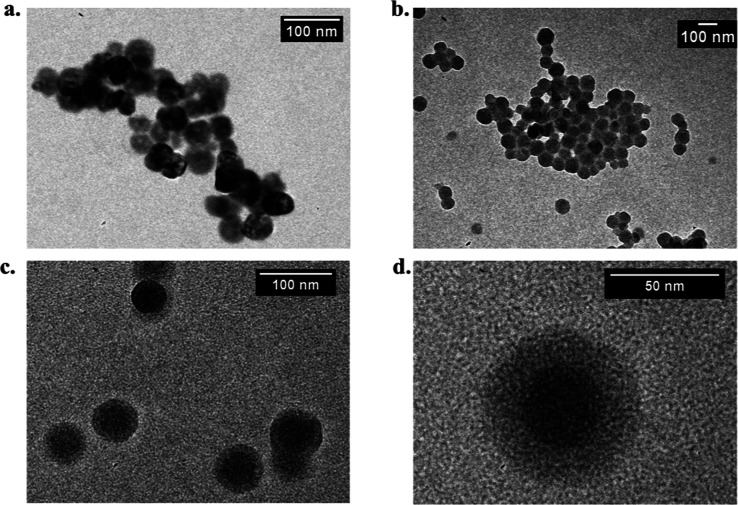
Transmission Electron Microscopy (TEM) images of (a) the UCNP coated with PAA, (b) ZIF-8 nanoparticles, (c) and (d) UCNP@ZIF-8 core–shell nanoparticles.

The Transmission Electron Microscopy (TEM) image of the modified UCNPs with PAA is shown in Figure 2a. The orthorhombic morphology of ZIF-8 nanoparticles with an average diameter of 61.30 ± 3.47 nm is illustrated in Figure 2b. Additionally, after ZIF-8 coating, the core–shell structure becomes evident, as shown in Figure 2c. The final average size of the UCNP@ZIF-8 particles was approximately 74.54 ± 4.72 nm. Complementary statistical analysis of the size distribution for UCNP-PAA, ZIF-8, and UCNP@ZIF-8 is provided in Figure S2 of the Supporting Information.

As depicted in the beautiful image shown in Figure 2d, the UCNP@ZIF-8 image at higher magnification clearly reveals the core–shell structure, where the shell site with 20 nm thickness is formed by ZIF-8, and the core is composed of UCNP with an average size of 35 nm.

**Figure 3:**
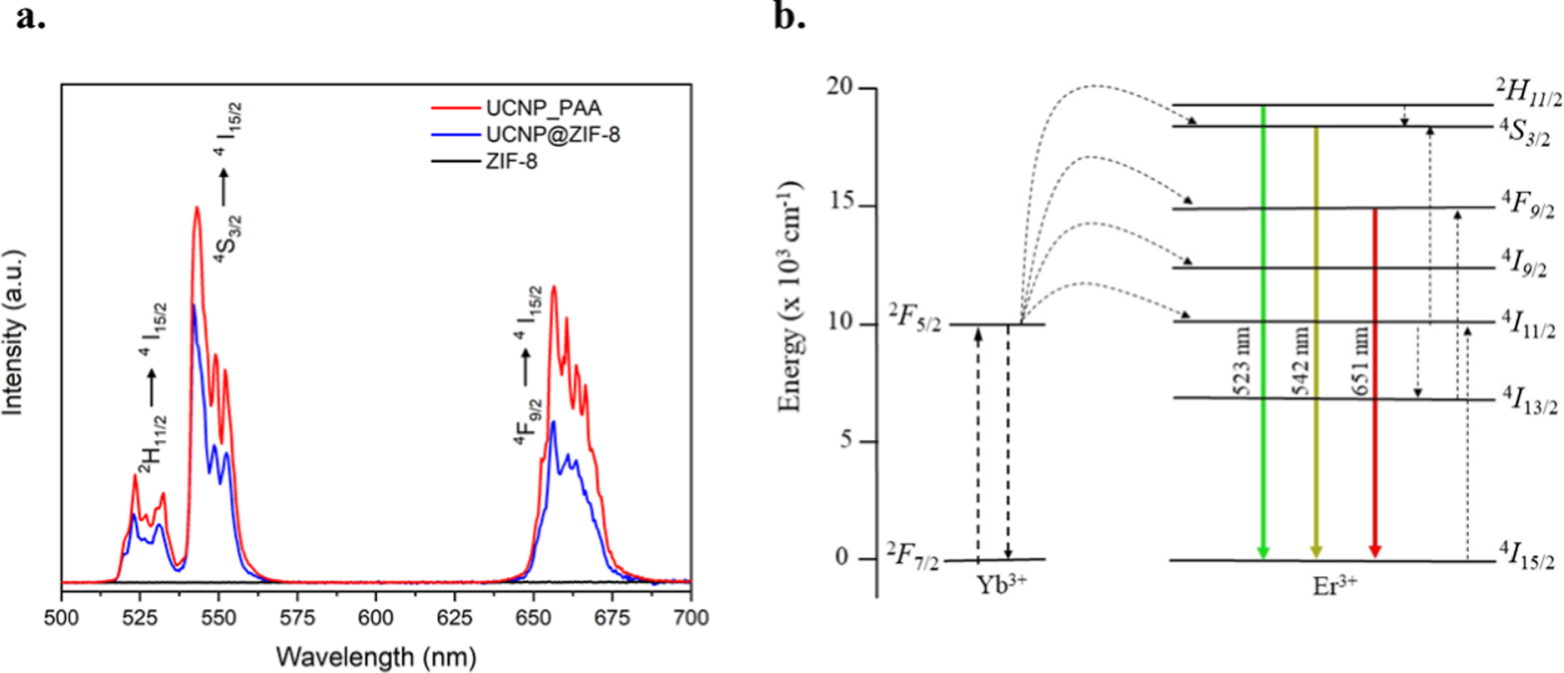
(a) Emission spectra of UCNP_PAA (red line), ZIF-8 (black line), and UCNP@ZIF-8 (blue line) nanoparticles, using λ_exc_ = 980 nm and power energy of 200 mW cm^–2^ (b) Schematic illustration of electronic transitions in UCNPs.

By fluorescence spectroscopy, the upconversion process of 10 mg mL^–1^ NaYF_4_/Yb^3+^Er^3+^ in water was investigated and analyzed for each nanoparticle system (UCNPs_PAA, ZIF-8, and UCNP@ZIF-8) using the infrared laser at 980 nm as an excitation source. As observed in the emission spectra of Figure 3a, three different emission regions with maximum intensity values at 520, 550 nm, and 650 nm are observed in the spectra. The highest energetic process is attributed to ^2^H_11/2_ → ^4^I_15/2_ transition, followed by others two lowest energetic processes which were attributed to ^4^S_3/2_ → ^4^I_15/2_, and ^4^F_9/2_ → ^4^I_15/2_, respectively. Notably, even after the formation of the core–shell structure with ZIF-8, the emission profile maintained the typical luminescent characteristics of the UCNPs, confirming that the encapsulation in ZIF-8 did not hinder the upconversion efficiency. See Figure 3b for the schematic electronic transitions illustrating the upconversion and emission processes.^21^

In summary, the characterization results confirm the successful formation of the core–shell UCNP@ZIF-8 structure. The FT-IR spectrum shows shifts and attenuation of bands, indicating interactions between PAA and ZIF-8. PXRD patterns reveal distinct reflections for the core–shell compared to the physical mixture, highlighting structural integration. The upconversion luminescence spectra confirm that UCNPs retain their optical properties without quenching in the physical mixture, further supporting the stability and structural integrity of the hybrid material. Comprehensive data, including PXRD patterns (Figure S3a), FT-IR spectra (Figure S3b), and luminescence spectra (Figure S3c) for the physical mixture, UCNP@ZIF-8, ZIF-8, and UCNPs (λ_exc_ = 980 nm), are presented in the Supporting Information.

### 3.2 Drug Loading and Release Studies

**Figure 4:**
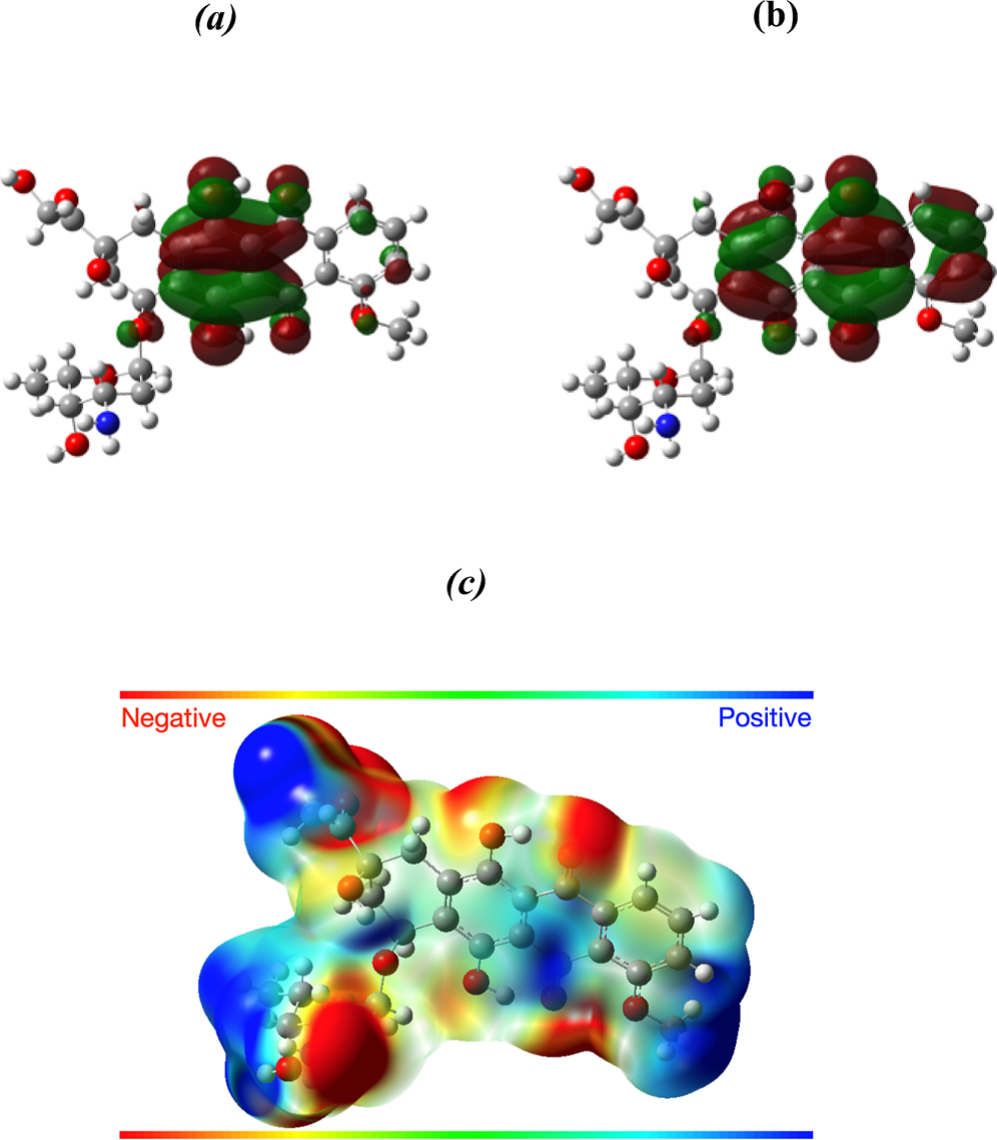
Representation of (a) HOMO, (b) LUMO orbitals, and (c) MESP for the DOX molecule.

Density Functional Theory (DFT) calculations examined the electrostatic interaction between doxorubicin (DOX) and the system. The chemical structure of DOX, depicted in Figure S4, highlights its key functional groups, such as amino, hydroxyl, carbonyl, and methoxy groups, which are crucial for potential electrostatic interactions. This structural representation provides insight into the molecular features responsible for binding and interaction behavior considered in the computational study. and the UCNP@-ZIF-8 structure. The goal was to gain insights into the adsorption and desorption processes observed in the experimental UV–vis measurements. The Molecular Electrostatic Surface Potential (MESP) was utilized to predict the electrostatic potential distribution across the entire density surface of the DOX molecule. Figure 4a–c shows the HOMO (Highest Occupied Molecular Orbital) and LUMO (Lowest Unoccupied Molecular Orbital) orbitals, along with the MESP distribution of the DOX molecule, respectively. The MESP map employs a color scale to represent electrostatic potential values: red areas indicate regions of negative potential, which correspond to nucleophilic centers, while blue areas signify positive potential, highlighting electrophilic centers.

According to the MESP results, the distribution of partial negative and positive charges throughout the DOX molecule suggests that the interaction that retains DOX within the UCNP@ZIF-8 structure is likely enhanced by the negative electrostatic potential located in the central region of the molecule. As shown in Figure 4a, the primary electrons in the HOMO of the DOX molecule are indeed localized in this central area, which may facilitate the entrapment of DOX by the positively charged sites of the ZIF-8 structure. However, the presence of positive electrostatic potentials in the DOX molecule indicates that this interaction might not be very strong, potentially leading to a destabilizing effect on the entrapment. This suggests that a gradual release of the DOX molecule from the UCNP@ZIF-8 structure is plausible. When examining the LUMO orbital (Figure 4b), it is observed that approximately 35% of the electron density from the HOMO orbitals is delocalized across the aromatic ring structures. This indicates a quenching of the negative electrostatic character in the LUMO orbital, resulting in a decreased interaction energy that helps keep the DOX molecule adsorbed within the nanoparticle structure. Therefore, upon excitation of the DOX molecule with visible light (specifically at wavelengths between 450 and 500 nm), the LUMO orbitals become populated, which should allow for the release of the DOX molecule from the nanoparticle structure.

**Figure 5:**
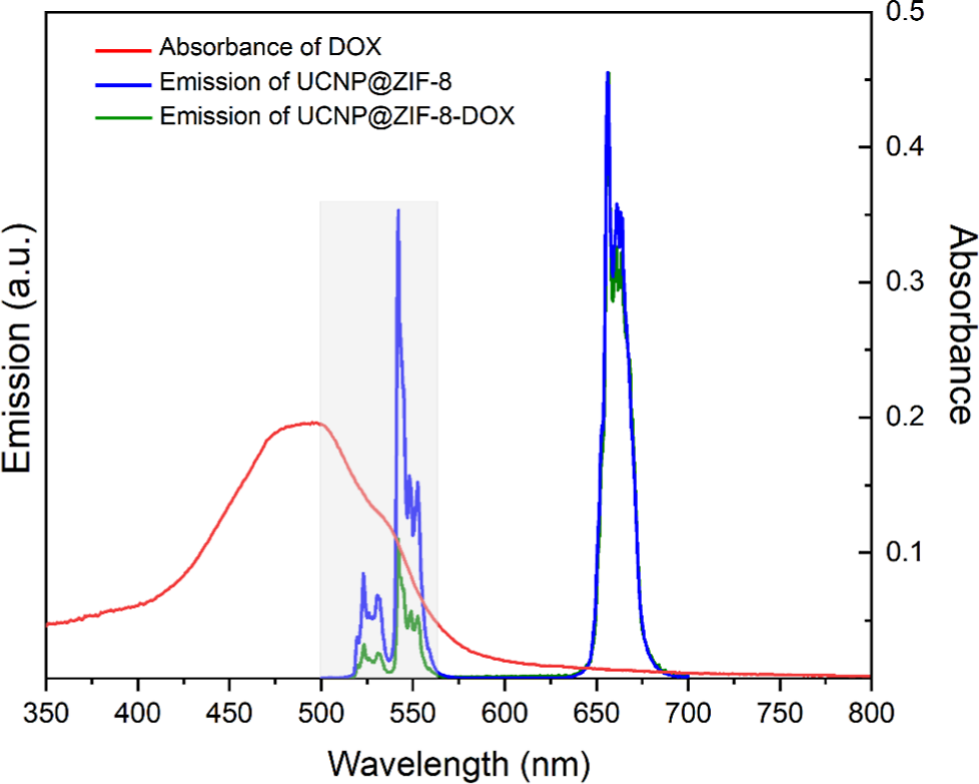
DOX UV–visible absorption spectrum (red line) and upconversion luminescence emission spectra of solutions of UCNP@ZIF-8 (10 mg mL-1, blue line) and UCNP@ZIF-8- DOX (10 mg mL-1, green line) in water, using λ_exc_ = 980 nm and power energy of 200 mW cm^–2^.

Figure 5 shows the emission spectrum of the UCNP@ZIF-8-DOX system when excited at 980 nm, compared to the emission of UCNP@ZIF-8 without DOX. As expected from the overlap between the DOX absorption spectrum and the emission features of the UCNP@ZIF-8 excitation at 980 nm led to a quenching of the emission bands where DOX absorbs (decrease the intensity in the 520–560 nm range), whereas the red emission was nearly unchanged. This suggests a direct interaction between the UCNP emission and the DOX absorption. The absence of a new emission band implies that the transferred energy does not produce significant emission from DOX, likely due to its low fluorescence under these conditions. This reduction in the UCNP@ZIF-8-DOX system’s emission intensity supports the role of DOX as an energy acceptor, consistent with the interaction mechanism proposed by DFT calculations. These experimental findings support the idea that energy transfer from UCNP to DOX leads to emission quenching, providing a foundation for studies on controlled DOX release under specific conditions.

The morphology and structure of the nanoparticles remained stable and intact following the incorporation of doxorubicin (DOX), as evidenced by the scanning electron microscopy (SEM) images presented in Figure S5. The micrographs show uniformly dispersed UCNP@ZIF-8-DOX nanoparticles with well-defined spherical shapes and smooth surfaces, indicating that the DOX encapsulation process did not alter their physical integrity. No significant aggregation or morphological deformation was observed, confirming the preservation of the structural characteristics after drug loading.

**Figure 6:**
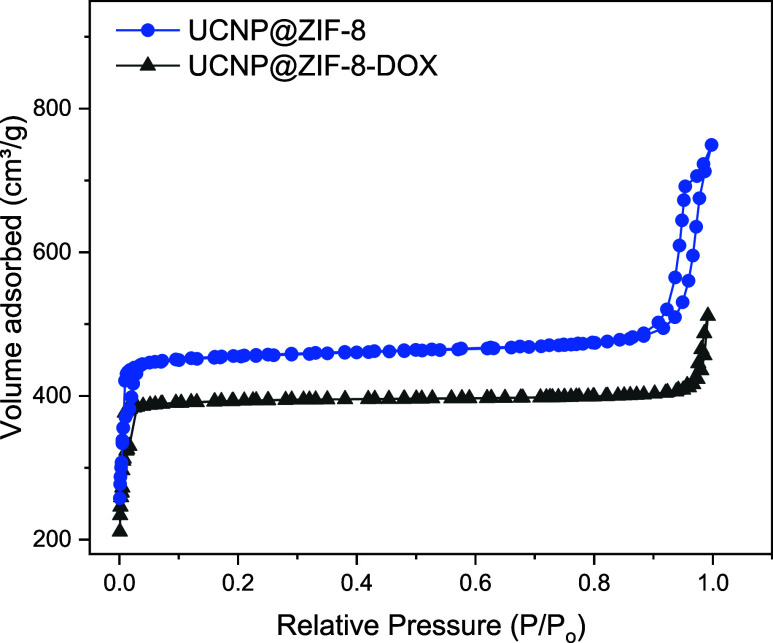
Nitrogen adsorption–desorption isotherms of UCNP@ZIF (in blue) and UCNP@ZIF-8-DOX (in black) nanoparticles.

As expected, due to the occlusion of the drug molecules, there was a decrease in the volume of the mesopores of zinc(II) MOF. The adsorption–desorption isotherms of UCNP@ZIF-8 core–shell composite, before and after loading the DOX are shown in Figure 6. Table S1 points to a decrease in the surface area from 1568 to 1352 m^2^ g^–1^ and pore volume from 0.74 to 0.66 cm^3^ g^–1^ after loading the drug.

As a typical antitumor drug, DOX was selected to evaluate the release behavior from drug-loaded UCNP@ZIF-8. In the DOX loading process, the DOX molecules are entrapped within the porous structure by an impregnation process. The aliquots were analyzed by UV–vis spectroscopy, and the drug was quantified using the calibration curve in methanol (*y* = 0.03*x* + 0.041). The drug loading capacity is determined to be 24.7 g g^–1^ (DOX/UCNP@ZIF-8), and the drug loading efficiency is 68 wt %.

Experiments were conducted with these different parameters to assess the influence of pH variation and laser action in the near-infrared region (980 nm) during the release process. It is important to highlight that the choice of power density used in the release assay was based on a previous study on MCF-7 tumor cells, where different power densities were tested at various contact times to evaluate the optimal parameter at which the laser alone would not cause cell death. Thus, the study of drug release influence could be conducted rather than focusing on cell death due to the direct effect of the laser. To achieve this, MCF-7 breast cancer cells (1 × 10^6^ cells per well) were plated in 96-well plates. Subsequently, the cells were cultured in RPMI 1640 medium and incubated in a humidified chamber for 24 h (37 °C, 5% CO_2_).

The cells were irradiated under different power densities, 0.2; 0.5; 0.8; 1, and 2 W cm^–2^, for 1, 3, and 5 min for each tested power, to assess the effect of NIR laser (980 nm) on cell viability. The effect of NIR laser (980 nm) on the cell viability of MCF-7 is shown in Figure S6. Over 95% of MCF-7 cells survived after irradiation with NIR laser at power densities of 0.2, 0.5, and 0.8 W cm^–2^. However, when the power density was increased to 1 W cm^–2^ during 5 min of irradiation, the cell viability decreased to 67%, which may indicate a hyperthermia process triggered by the heat process generated by laser exposition at higher power intensity. Based on these findings, a lower power intensity of 0.8 W cm^–2^ of the laser source was used in our irradiation measurements. The absence of irradiation and the effect of different power intensities of the laser source at 980 nm was also investigated in the drug release process.

The in vitro drug release assay is related to the process by which the drug is released from its matrix and becomes available in physiological mediums, serving as an initial and crucial tool for the development of new drug delivery systems. According to Rui Manadas and colleagues, these assays allow for the initial evaluation of release profiles for future actions aimed at optimizing the efficacy and safety of the system and correlating the in vitro and in vivo profiles, thus establishing parameters in an attempt to predict the in vivo behavior of the system.^22^ In all these experiments, Doxorubicin release in the free form was studied by UV–vis’s analysis.

The drug released from the studied material, which was proportionally related to the amount of drug present in the receptor medium, was calculated from the analytical curve of DOX in the physiological medium at pH 7.4 and pH 5.0, simulating the health medium and the acidic environment of the interstitial site of tumor cells, respectively.^23^ Additionally, a study was conducted on the release profile of DOX at pH 5 with the application of a 980 nm laser for 5 min and a power density of 0.8 W cm^–2^.

The analytical curves of DOX yielded the following line equations: in PBS at pH 7.4, *y* = 0.002*x* – 0.0034 with a correlation coefficient (*R*^2^) of 0.990; and in PBS at pH 5.0, *y* = 0.013*x* + 0.0116 with an *R*^2^ of 0.992. These high correlation coefficients indicate the linearity of the curves.

**Figure 7:**
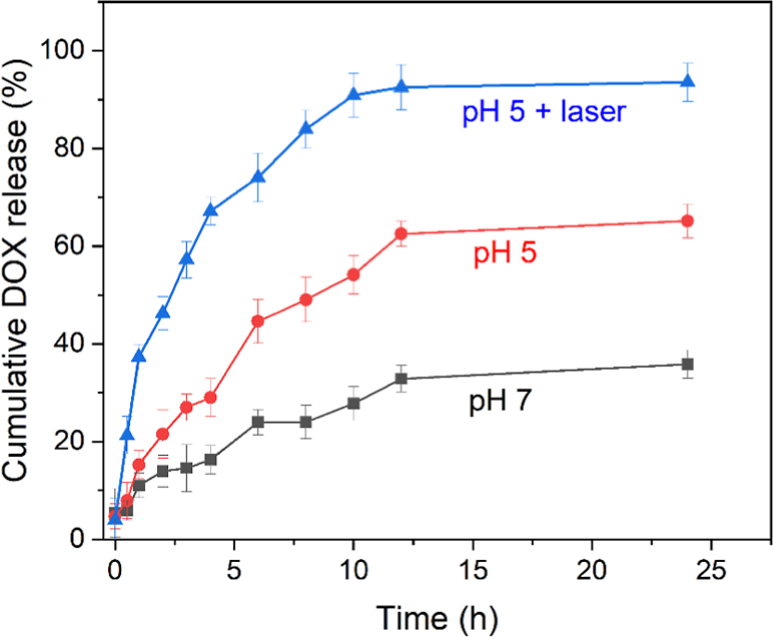
Release profiles of DOX with the effect of NIR laser (980 nm).

The drug release profiles from the UCNP@ZIF-8-DOX core–shell nanoparticles at different pH values and with Laser 980 nm influences are shown in Figure 7.

The release profiles indicated that about 37% of DOX was released from the UCNP@ZIF-8 nanoparticles after 24 h in a receptor medium with a pH of 7.4, suggesting that the drug is adsorbed in the pores of ZIF-8 through coordination interactions. Additionally, 62% of DOX was released within 12 h in an acidic receptor medium, demonstrating a significantly higher pH-responsive release capacity. The release mechanism of the core–shell structure is distinct from conventional nanoparticles. Specifically, the release of DOX from UCNP@ZIF-8 in an acidic medium is linked to the dissociation of the ZIF-8 “shell”. This suggests that drug release in a normal physiological medium is minimal, with most of the release occurring under acidic conditions after internalization in the tumor microenvironment. Therefore, UCNP@ZIF-8-DOX nanoparticles hold promise as pH-responsive drug delivery systems for cancer therapy. It is important to highlight that the crystalline structure of the core–shell UCNP@ZIF-8 remained intact under acidic conditions and after exposure to the 980 nm laser, as demonstrated in Figure S7. This stability emphasizes the robustness of the core–shell system, ensuring that the laser’s effect is limited to modulating drug release without compromising the structural integrity of the carrier.

Furthermore, an initial rapid release of DOX is preferable to eliminate tumor cells at the outset. Subsequently, a sustained release of DOX is essential to inhibit the further proliferation of any remaining tumor cells. This dual-phase release mechanism ensures that both the initial and residual cancerous cells are effectively managed, enhancing the overall efficacy of the treatment. By combining these release strategies, the UCNP@ZIF-8-DOX nanoparticles provide a comprehensive approach to cancer therapy, optimizing both immediate and long-term therapeutic outcomes.

After the incidence of a 980 nm laser, the DOX release in a pH 5 receptor medium solution was 90% after 12 h, demonstrating a significant enhancement in drug release. The increase in temperature caused by the laser can be an important factor in controlling the drug release rate. The absorption of the laser in the near-infrared region causes local hyperthermia, contributing to the diffusion of the drug in the system. It is important to note that a prior test confirmed that the power density and duration of the applied laser do not cause cell death; therefore, the laser temperature also did not influence the treatment. Additionally, when excited, the upconversion nanoparticles emit light in the drug’s absorption region, potentially altering its structural conformation and release profile. For this reason, UCNP@ZIF-8-DOX nanoparticles are promising candidates for pH and NIR laser-responsive drug delivery systems in cancer therapy.

Considering that the emission band of the UCNP in the green region aligns with the excitation wavelength range of DOX, corresponding to visible light (blue-green), it can be posited that utilizing a NIR laser to excite the UCNP induces a cascade effect, subsequently exciting the DOX. This interaction may alter the conformation of the DOX molecule, thereby facilitating a greater release of the compound in response to the laser stimulus. Additionally, the activation of excited states of DOX through light or energy transfer processes reveals that the electron distribution in the central region of the DOX structure diminishes in the LUMO compared to the HOMO. This discrepancy suggests that the excited state of DOX also plays a role in destabilizing the entrapment process. Both scenarios (involving HOMO or LUMO) indicate that DOX molecules could be released gradually over time, aligning with the anticipated behavior of controlled release systems.

The kinetic DOX release profiles were fitted using mathematical models (Sigma Plot 10.0 software-Systat Software, San Jose, CA, USA): Korsmeyer–Peppas, Higuchi, Hixson–Crowell, first order, and Weibull. Table 1 provides an overview of the drug release models and their corresponding fitting equations and regression parameters for each model.

**Table 1:** Kinetic Models Were Applied to Analyze the DOX Release from UCNP@ZIF-8, and Their Corresponding Calculated Parameters.

	first order	Higuchi	Korsmeyer–Peppas		Hixson–Crowell	Weibull	
	*R*^2^	*R*^2^	*R*^2^	η	*R*^2^	*R*^2^	*b*
pH 7	0.63	0.94	0.98	0.400	0.54	0.96	9.88
pH 5	0.91	0.95	0.99	0.469	0.86	0.96	7.13
pH 5 with laser	0.97	0.85	0.99	0.497	0.91	0.97	3.34

The drug release kinetics were analyzed through various models to understand the underlying mechanisms. The Korsmeyer–Peppas model was used to describe more complex release mechanisms involving a combination of drug diffusion, matrix swelling, and surface erosion.^24^ Additionally, the Hixson-Crowell model was employed to study surface erosion, focusing on how the material disintegrates into smaller fragments during the release process.^25^ Zero-order kinetics was applied to assess diffusion-controlled release, where the drug is released at a constant rate, indicative of slow, sustained release over time.^26^ For systems involving hydrolytic degradation, the first-order kinetic model was used, as it effectively characterizes the rate of degradation and drug release based on concentration-dependent behaviors.^27^

The DOX release from the UCNP@ZIF-8 nanoparticles at both pH values strongly correlated with the Korsmeyer–Peppas model. The drug release profile of doxorubicin from the UCNP@ZIF-8 system was modeled using the Korsmeyer–Peppas equation, with η values providing insight into the release mechanism. At pH 7 (η = 0.4), the release is primarily governed by Fickian diffusion, where the value of η ≤ 0.45 indicates that diffusion controlled the release process, resulting in a controlled and slow release. At pH 5 (η = 0.469), the release remains diffusion-driven, showing a slightly more efficient release due to the partial degradation of the matrix in the acidic environment. Under pH 5 with 980 nm laser stimulation (η = 0.497), the η value suggests a faster, more complex release mechanism that approaches the range of anomalous or non-Fickian transport, where η values are between 0.45 and 0.89. This indicates a combination of diffusion, degradation, and external stimuli that collectively influence drug release. These results demonstrate that drug release can be modulated by both pH and laser irradiation, highlighting the potential of the system for controlled and stimulus-responsive therapies.

### 3.3 Cell Culture, Viability Evaluation, and Cellular Analysis

MCF-7, SKBr, and MCF-10 cells were treated with different concentrations (0–300 μg mL^–1^) of UCNP@ZIF-8 and UCNP@ZIF-8-DOX nanoparticles for 48 h, and then an MTT assay was performed to evaluate the cell viability. The graphs of cell viability of UCNP@ZIF-8, UCNP@ZIF-8-DOX, and DOX nanoparticles are shown in Figure S8.

The nanoparticle without the drug, 300 μg mL^–1^, the highest concentration used, demonstrated percentages of viable cells above 70%, suggesting that there is no cytotoxicity of the developed nanoparticles, except the SKBr cell line, which presented cellular viability of 40% at 300 μg mL^–1^, showing some selectivity for this cell line.

Statistical analysis of the inhibitory concentrations of 50% of viability (IC_50_) was also performed. Table 2 summarizes the obtained IC_50_ values.

**Table 2:** IC_50_ Values (in μg mL^–1^) for UCNP@ZIF-8 and UXNP@ZIF-8-DOX Nanoparticles in MCF-10, MCF-7, and SKBr Cell Lines.

	MCF-10A	MCF-7	SKBr
DOX	0.372 ± 0.68	0.698 ± 0.33	0.245 ± 0.46
ZIF-8	257.04 ± 1.26	246.08 ± 1.64	239.88 ± 1.02
UCNP	>300	>300	>300
UCNP@ZIF-8	>300	>300	292.41 ± 0.62
UCNP@ZIF-8-DOX	143.25 ± 1.39	121.62 ± 0.49	112.94 ± 0.34

The cytotoxicity analyses tested showed that the developed compound UCNP@ZIF-8 presented greater cytotoxicity for the SKBr tumor cell line, with an IC_50_ value of 292.41 μg mL^–1^, while for the MCF-7 tumor cell line and the healthy MCF-10A cell line, an IC_50_ value of >300 μg mL^–1^ was observed in both cell lines after 48 h of treatment.

The compound’s activity containing the incorporated drug doxorubicin showed some selectivity for the tumor cell line, compared to the healthy MCF-10 cell line, thus being more cytotoxic for the tumor cell lines studied.

To study the stimuli-responsive capacity of the laser for cytotoxicity, the cell viability assay was performed with the application of the NIR laser (980 nm) for 5 min at a power density of 0.8 W/cm^2^, based on the study previously performed and presented in Figure S6. Besides that, considering that the effect of the laser for higher drug release and greater efficiency will be mainly evaluated, the experiments were carried out with tumor (MCF-7) and healthy (MCF-10) breast cell lines.

**Figure 8:**
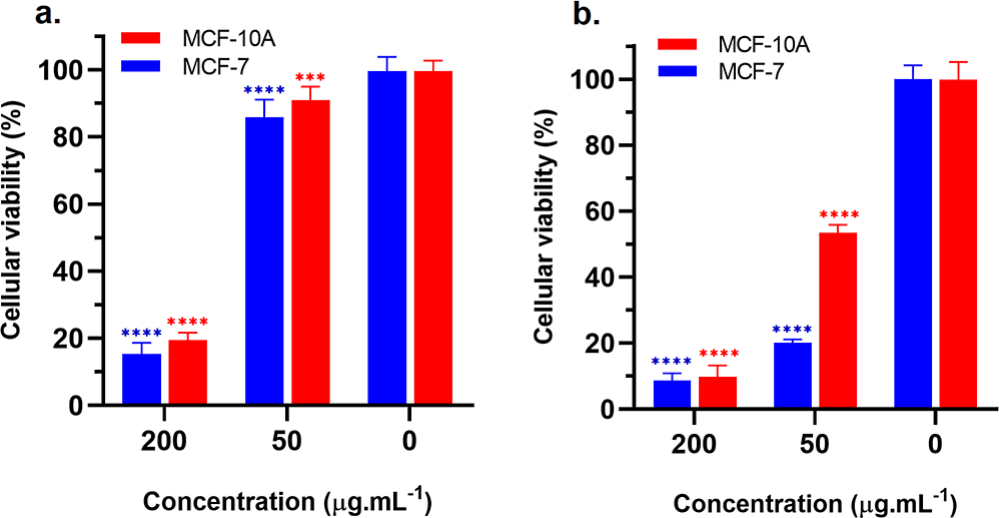
Cell viability of MCF-7 and MCF-10A cells after 48 h of incubation with UCNP@ZIF-8-DOX (a), and UCNP@ZIF-DOX nanoparticles with 980 nm laser application (b) at different concentrations, measured by MTT assay (****P < 0.0001; ***P < 0.001 versus control).

The cell viability of the tumor cell line (MCF-7) and the healthy breast cell line (MCF-10A) in contact with the developed compound UCNP@ZIF-8-DOX and with the application of the laser is shown in Figure 8.

Figure 8 showed that after laser application, cell viability decreased significantly at a concentration of 50 μg mL^–1^, from 90.9% to 53.5% when applied to the healthy cell line (MCF-10A) and from 85.7% to 20% when applied to the tumor cell line (MCF-7).

Therefore, the developed core–shell material UCNP@ZIF-8-DOX demonstrated responsiveness to laser irradiation, which is a promising feature for cancer treatment as it enables selective activation of the antitumor drug. This selectivity can enhance therapeutic effectiveness while reducing adverse effects. The lower toxicity observed for the UCNP@ZIF-8-DOX system compared to free DOX can be attributed to the advantages of controlled drug release systems. One of the key benefits is the ability to modulate drug release through external stimuli, such as pH changes and laser irradiation. This controlled release strategy maintains a lower concentration of free drug in the bloodstream at any given time, thus reducing rapid exposure to high doses that are typically associated with the systemic toxicity of chemotherapy. By allowing gradual and localized drug release, the system minimizes peak concentrations of free DOX, potentially reducing its metabolism and elimination rates. This modulation helps mitigate common adverse effects, such as cardiotoxicity, and improves the overall safety profile of the treatment. Together, these findings highlight the potential of the UCNP@ZIF-8-DOX system as a stimulus-responsive drug delivery platform for cancer therapy.

**Figure 9:**
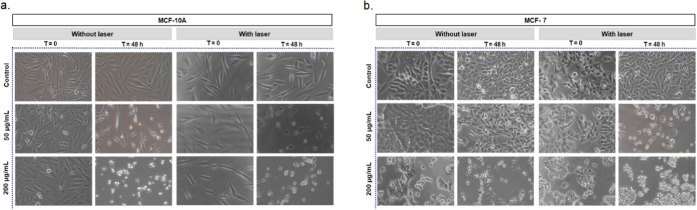
Effect of UCNP@ZIF-DOX nanoparticles on cell morphology in the nonbreast tumor cell line MCF-10A (a), and breast tumor cell line MCF-7 (b) without and with the application of 980 nm laser. The experiment was performed in triplicate. The images correspond to one of the representative triplicates of each cell line.

The cell morphology assay was performed to qualitatively evaluate the effect of the UCNP@ZIF-8-DOX core–shell on the MCF-7 breast tumor cell line and the MCF-10A nontumor breast cell line with and without the application of the 980 nm laser. This assay was performed to, together with the cytotoxicity assay, show the effect of the developed system in cellular medium, as shown in Figure 9.

At 48 h, at the lowest concentration tested, cell shrinkage and the formation of some circular structures were observed, probably indicating the beginning of cell death. On the other hand, in cells treated with 980 nm laser, a greater decrease in cell density, formation of partially detached circular structures, and cell shrinkage were observed, indicating more cell death at the same concentration of the developed compound.

The study of cell morphology corroborates the cell viability assay, evidencing greater cytotoxicity when the developed compound is associated with 980 nm laser.

The colony formation assay, or clonogenic assay, is an in vitro cell survival test based on the ability of a single cell to grow and reproduce into colonies. It is used to evaluate the reproductive capacity of cells after exposure to cytotoxic agents, such as certain chemotherapeutics. This assay allows for the observation of cytotoxic effects (decrease in the number of colonies) and cytostatic effects (decrease in the size of colonies) of the combination on the proliferative ability of the MCF-10A and MCF-7 cell lines.

**Figure 10:**
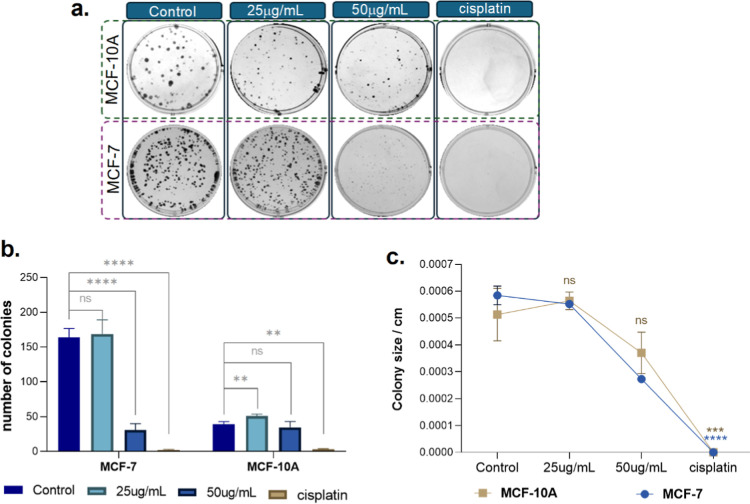
Clonogenic Assay: (a) Image of the colony formation assay plates showing the effect of UCNP@ZIF-8-DOX on tumor cells (MCF-7) and nontumor cells (MCF-10A) of the breast. Graphs of (b) number of colonies and (c) colony size in both cell lines. The data are expressed as mean ± SEM ****P < 0.0001, ***P < 0.001, **P < 0.01, and *P < 0.1 in one-way ANOVA followed by Dunnett’s multiple comparison tests between control and treated cells at each concentration.

In Figure 10, both the control and cells treated with 25 μg mL^–1^ of the nanomaterial UCNP@ZIF-8-DOX exhibited comparable colony counts in the MCF-7 and MCF-10A breast cell lines. This confirms the viability data, which suggests that the material has no cytotoxic effects at this concentration. In contrast, cells treated with concentrations of 50 and 100 μg mL^–1^ of UCNP@ZIF-8-DOX inhibited growth and colony formation in both cell lines, demonstrating the cytotoxic effect of the nanomaterial at high concentrations (Figure 10b). At the same concentrations, a cytostatic effect was also observed, resulting in a decrease in colony size in both lines (Figure 10c), as indicated by the statistical significance data.

## 4 Conclusion

In summary, the development of a multifunctional theranostic material based on upconversion nanoparticle (UCNP) and type-MOF ZIF-8 porous crystalline materials was proposed in this work, which can be used together for imaging and controlled drug release of pH and stimulus-responsive laser. The surface of the upconversion nanoparticle was modified with PAA and made up the core of the structure in which the shell is formed by ZIF-8 crystal. The MOF was able to incorporate a large amount (∼ 70 wt %) of the antitumor doxorubicin forming a UCNP@ZIF-DOX composite. Under the action of the 980 nm laser, the release profile of DOX in the acidic pH buffer solution reached 90%. This result shows that the laser significantly increased the release of the drug. The activity of the compound containing the incorporated drug doxorubicin was selective for the tumor cell line when compared to the healthy cell line, thus being more cytotoxic to the tumor cell lines studied. Furthermore, after application of the 980 nm laser, cell viability decreased significantly (concentration of 50 μg mL^–1^ of UCNP@ZIF-8 with DOX), going from 85.7% to 20% when applied to the tumor cell line (MCF-7). The clonogenic assay indicates that the nanomaterial exhibits cytotoxic and cytostatic characteristics at higher concentrations of 50 and 100 μg mL^–1^. The ability to respond to pH and laser makes this material a promising system for cancer treatment, allowing for selectivity of action of the antitumor drug, increasing its therapeutic effectiveness, and reducing the adverse effects that this class of medication can cause to cancer patients.

## Supporting Information Available

Additional figures, tables, and methods related to the study are provided (PDF)
