# Supplementary material for: Core–Shell UCNP@MOF Nanoplatforms for Dual Stimuli-Responsive Doxorubicin Release
Source: ACS Appl Bio Mater. 2025 Apr 9;8(4):2954–64. doi: 10.1021/acsabm.4c01796 (PMC12015952; doi:10.1021/acsabm.4c01796)
Supplement: Supplementary file 1 — mt4c01796_si_001.pdf [file mt4c01796_si_001.pdf]

Supporting information

Core-shell UCNP@MOF nanoplatforms for dual stimuli-responsive doxorubicin release

Marina P. Abuçafy[a,*], *Beatriz B. S. Ramin*[a], *Angelica E. Graminha*[a], *Willy G. Santos*[a,b], *Regina C. G. Frem*[a], *Adelino V. G. Netto*[a], *José Clayston M. Pereira*[a], *Sidney J. L. Ribeiro*[a,*]

[a] São Paulo State University, Institute of Chemistry, Araraquara, SP, 14800-060, Brazil

[b] Federal University of ABC, UFABC, Santo André, SP, 09210-170, Brazil

Corresponding authors: marina.abucafy@gmail.com; sidney.jl.ribeiro@unesp.br

Contents of Supporting Information Files

Figure S1: Zeta potential values of UCNP-PAA, ZIF-8, and UCNP@ZIF-8 nanoparticles measured by dynamic light scattering (DLS) technique, showing the surface charge of each nanoparticle type.

Figure S2: Statistical analysis of the size distribution of (a) UCNP-PAA, (b) ZIF-8, and (c) UCNP@ZIF-8 nanoparticles, indicating the particle size range for each sample.

Figure S3: (a) PXRD patterns, (b) FT-IR spectra, and (c) luminescence spectra (excited at 980 nm) of the physical mixture, core-shell UCNP@ZIF-8 composite, ZIF-8, and UCNPs, providing information on the structural and optical properties of the materials.

Figure S4: Chemical structure of the doxorubicin (DOX) molecule, showing its molecular composition and key functional groups.

Figure S5: Scanning electron microscopy (SEM) images of UCNP@ZIF-8-DOX nanoparticles, displaying the morphology and surface characteristics of the nanoparticles.

Figure S6: Effect of NIR laser (980 nm) on the cell viability of MCF-7 cells at various power densities (0, 0.2, 0.5, 0.8, 1, and 2 W cm^{-2}) and different contact times (1 min, 3 min, and 5 min), illustrating the cytotoxic effects under laser irradiation.

Figure S7: PXRD patterns of the core-shell UCNP@ZIF-8 nanoparticles before and after exposure to acidic conditions and 980 nm laser irradiation, showing any structural changes under these conditions.

Figure S8: Cell viability of MCF-7, SKBr, and MCF-10A cells after 48 hours of incubation with UCNP@ZIF-8 (a), UCNP@ZIF-DOX (b), and free DOX (c) nanoparticles at different concentrations, assessed by MTT assay to evaluate the cytotoxicity of the nanoparticles.

Table S1: Textural properties of UCNP@ZIF-8 and UCNP@ZIF-8-DOX nanoparticles, including specific surface area, pore volume, and pore diameter, highlighting the differences in textural properties due to the drug encapsulation.

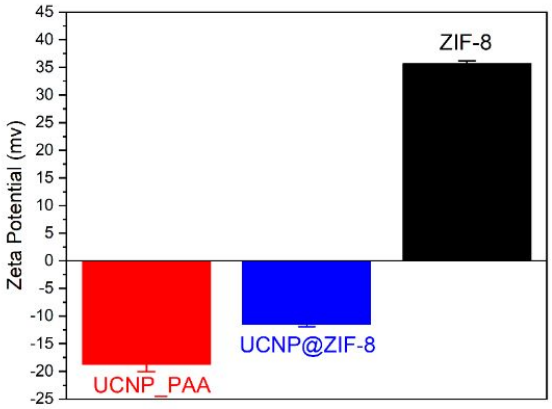

Figure S1. Zeta potential values obtained by dynamic light scattering technique for UCNP_PAA, ZIF-8, and UCNP@ZIF-8.

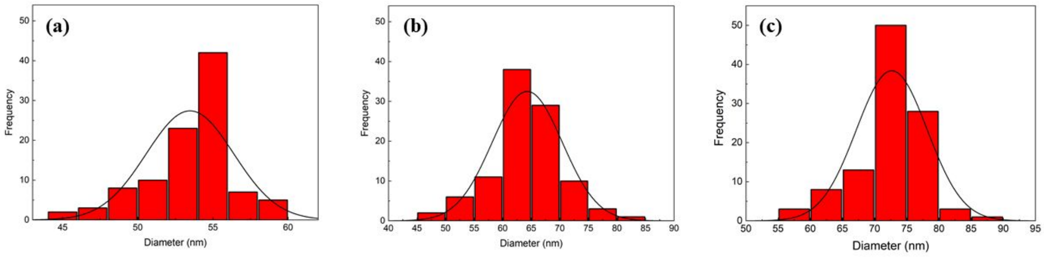

Figure S2. Statistical analysis of size distribution (a) UCNP-PAA, (b) ZIF-8 and (c) UCNP@ZIF-8.

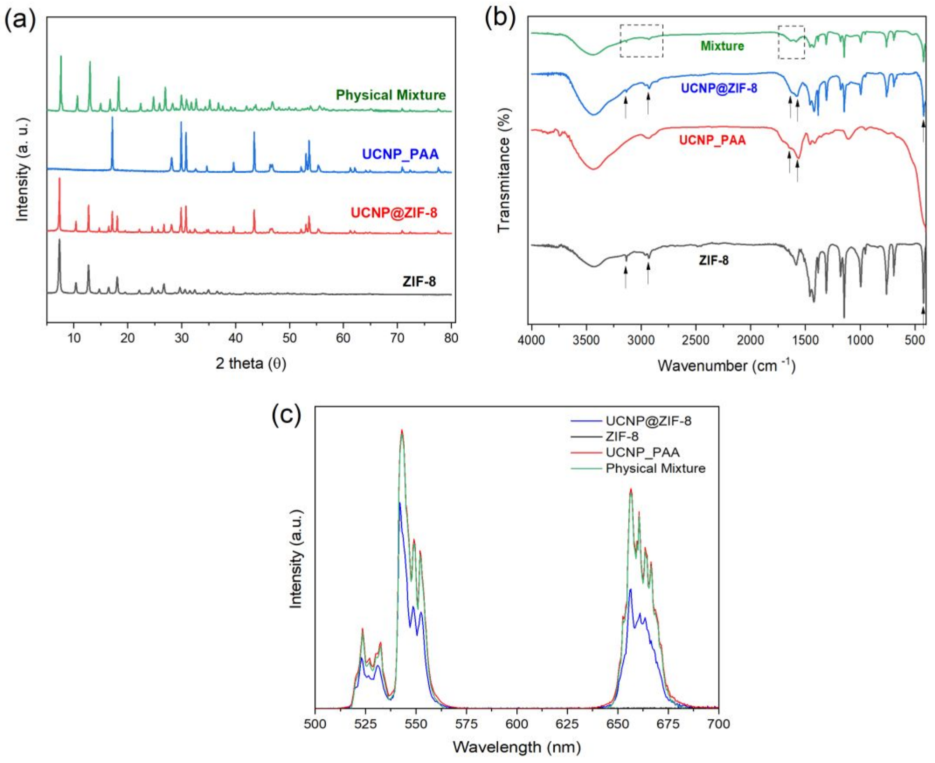

Figure S3. (a) PXRD, (b) FT-IR spectra patterns and (c) Luminescence spectra of the physical mixture, the core-shell UCNP@ZIF-8 composite, ZIF-8 and UCNPs (λ_{exc}= 980 nm).

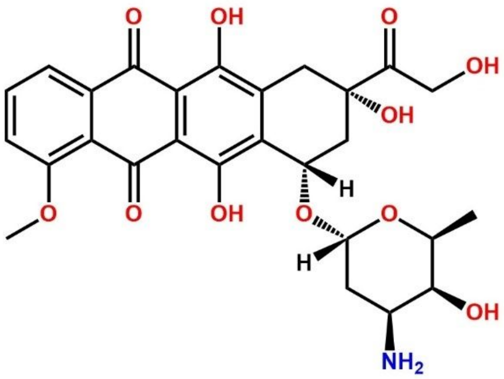

Figure S4. Chemical structure of the doxorubicin (DOX) molecule.

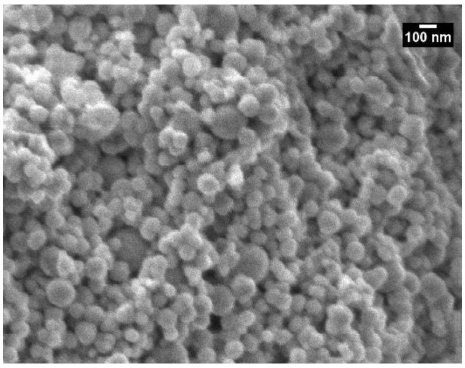

Figure S5. Scanning electron microscopy (SEM) images of UCNP@ZIF-8-DOX nanoparticles.

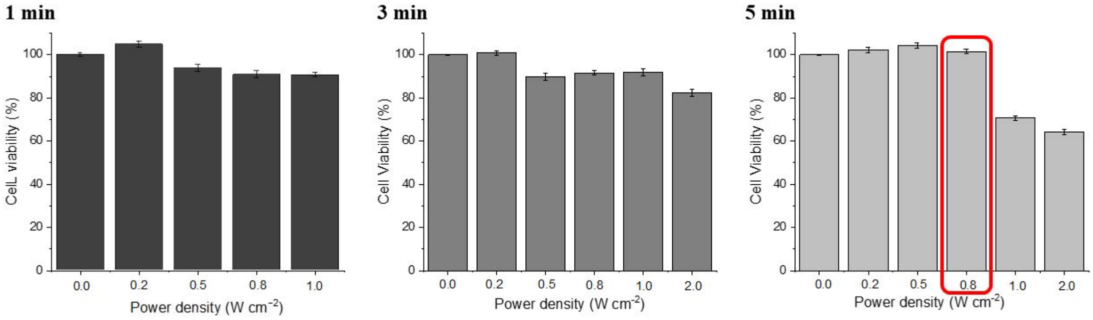

Figure S6. The effect of NIR laser (980 nm) on the cell viability of MCF-7 at different power densities (0, 0.2, 0.5, 0.8, 1, and 2 W cm^{-2}) and at different contact times (1 min, 3 min, and 5 min).

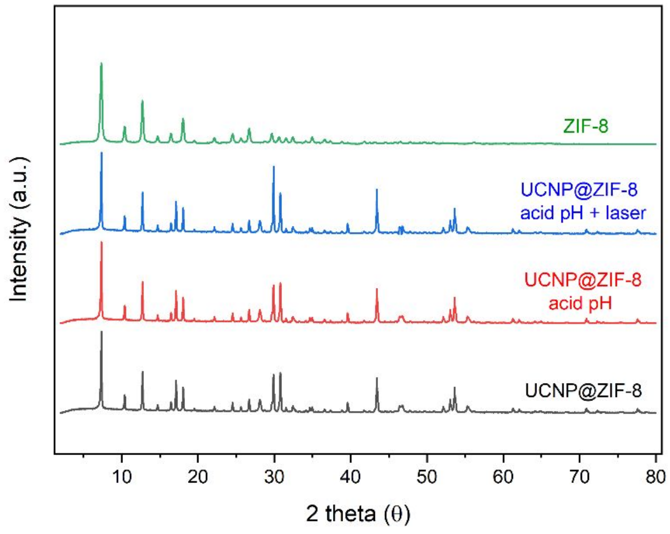

Figure S7: PXRD patterns of the core-shell UCNP@ZIF-8 before and after exposure to acidic conditions and 980 nm laser irradiation.

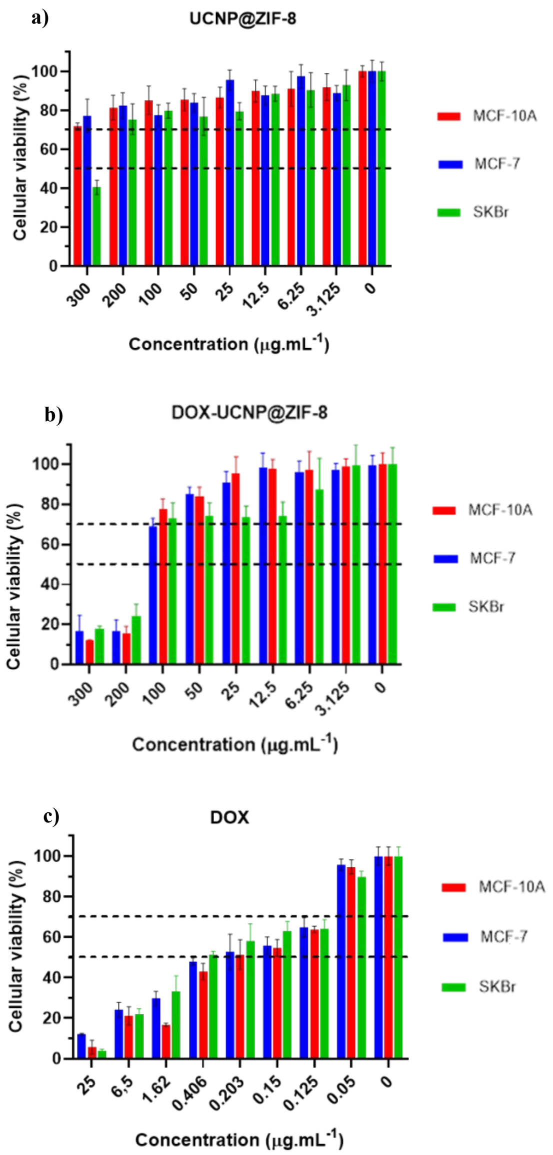

Figure S8: Cell viability of MCF-7, SKBr and MCF-10A cells after 48 h of incubation with UCNP@ZIF-8 (a), UCNP@ZIF-DOX (b) and DOX (c) nanoparticles at different concentrations, measured by MTT assay.

Table S1: Textural evaluation of UCNP@ZIF-8 and UCNP@ZIF-8-DOX nanoparticles.

**Sample**	**Specific surface area ($m^2.g^{-1}$)**	**Pore volume ($cm^3.g^{-1}$)**	**Pore diameter (nm)**
UCNP@ZIF-8	1568	0.74	2.02
UCNP@ZIF-8-DOX	1352	0.66	1.20
